# Medical Schools

**Published:** 1850-05

**Authors:** 


*Medical Schools.*—At the last dates there were thirty-five medical schools in the United States, viz: in Pennsylvania, five; in New York, five; in Maryland, two; in Massachusetts, two; in Vermont, two; in Virginia, two; in Kentucky, two; in Ohio, three; and in Missouri, four. In the other States, only one exists, or none, in each State. The oldest medical school in the United States is the old medical department of the University of Pennsylvania, which was established in 1762. Next, we have the medical school of Harvard College, founded in 1782, and that of Dartmouth in New Hampshire, established in 1797.

The oldest institution in the West, for medical instruction, is at Lexington, founded in 1818, and having now 1,351 graduates. The numbers of graduates at Pennsylvania University is 4,952: at Jefferson Medical College, 1,598; at Baltimore, 909; at the College of Physicians and Surgeons, New York, 852: at Yale, 850; at Louisville, 688; at the University of New York, 597; New Hampshire, 858; Maine medical school, 596, &c. The institutions having the largest classes at present, are the University of Pennsylvania, 508; Jefferson Medical College, 480; Medical Faculty at the New York University, 421; Louisville, 376; Cleveland, 259; the College of Physicians and Surgeons, New York, 219. The number of Professors in the several schools ranges from three to nine, viz: one has three, (University of Virginia); three have five; thirteen have six; thirteen have seven; three have eight; two have nine, (the St. Louis colleges.)—***Western Journal of Med. & Surgery.***

